# 3D escape: an alternative paradigm for spatial orientation studies in insects

**DOI:** 10.1007/s00359-022-01574-x

**Published:** 2022-10-03

**Authors:** Christoph Bruns, Susanna Labisch, Jan-Henning Dirks

**Affiliations:** Biomimetics-Innovation-Centre, Hochschule Bremen - City University of Applied Sciences, Bremen, Germany

**Keywords:** Gravitaxis, Biomechanics, Exoskeleton, Mechanoreceptors, Insects

## Abstract

Arthropods and in particular insects show a great variety of different exoskeletal sensors. For most arthropods, spatial orientation and gravity perception is not fully understood. In particular, the interaction of the different sensors is still a subject of ongoing research. A disadvantage of most of the experimental methods used to date to study the spatial orientation of arthropods in behavioral experiments is that the body or individual body parts are fixed partly in a non-natural manner. Therefore, often only the movement of individual body segments can be used to evaluate the experiments. We here present a novel experimental method to easily study 3D-escape movements in insects and analyze whole-body reaction. The animals are placed in a transparent container, filled with a lightweight substrate and rotating around two axes. To verify our setup, house crickets (*Acheta domesticus*) with selectively manipulated gravity-perceiving structures were analyzed. The spatial orientation behavior was quantified by measuring the time individuals took to escape toward the surface and the angular deviation toward the gravitational vector. These experiments confirm earlier results and therefore validated our experimental setup. Our new approach thus allows to investigate several comprehensive questions regarding the spatial orientation of insects and other animals.

## Introduction

For almost all organisms, spatial orientation due to a reliable detection of the gravitational vector is an important ability indirectly affecting their reproductive success. This detection is based on relatively simple physical principles such as masses moving relatively to each other, however, physiologically implemented using a variety of different sensor types (Markl 1963; Bender and Frye 2009).

Arthropods show a great variety of different exoskeletal sensors sensitive to the gravitational vector (Bender and Frye 2009). At least three methods of sensing gravity are known to exist in terrestrial insects. The first method involves specialized gravity receptors. The abdominal appendages, called cerci, of cockroaches (Walthall and Hartman 1980) and crickets (Bischof 1975; Horn and Bischof 1983) carry club-shaped sensilla. These sensilla have a thick liquid-filled end that acts like a pendulum, deflecting in the direction of the gravitational vector. This displacement is in turn sensed by campaniform sensilla in the cuticle to obtain information about the gravitational vector and for spatial orientation (Bischof 1975; Gnatzy and Schmidt 1971; Walthall and Hartman 1980).

Most insects, however, lack such specialized sensory organs. Instead, they perceive the relative deflection between body appendages. This second method uses, for example, distributed proprioception by hair plates in the joints between segments (Markl 1963; Matthews and Matthews 2009). These hair plates can be found in various joints throughout the body of all groups of insects and are primarily used to control movement (Jander et al. 1970). By deflecting the hair sensilla, information about the load and position of the joints is recorded. However, the insect is also able to differentiate and combine this information to determine gravity-induced deflection of body parts and, thus, also to orientate spatially (Bässler 1961, 1965; Markl 1963; Matthews and Matthews 2009; Tuthill and Wilson 2016; Tuthill and Azim 2018). So, it's more of a general sense of position than a true gravity receptor (Markl 1963), but not all proprioceptive hair plates contribute equally to gravity perception in every insect. The greatest importance is attributed to the hair plates in the leg joints (Bässler 1961, 1965; Markl 1963) and in the neck joint between the head and thorax, which is also known as the prosternal organ (Lindauer and Nedel 1959; Markl 1962, 1963; Bender and Frye 2009). Another sensory structure that uses this principle to perceive information about gravity and has been studied in flies is the Johnston organ, which is located in the second antennal segment (pedicel). The Johnston organ is a collection of internal mechanosensory neurons that act as strain receptors and are stimulated by vibration to sense the deflection of the third antennal segment (funiculus) relative to the second (Kamikouchi et al. 2006; 2009; Tuthill and Wilson 2016). Horn and Kessler (1975) provided evidence that in fly antennae the proprioceptive hair plates on the first antenna segment (scape) also contribute to gravity perception by measuring the deflection between the first and second antennal segments. The same could be observed for crickets (Horn and Bischof 1983).

In the third mechanism, campaniform sensilla are used to determine the load transfer among legs in cockroaches and stick insects. Campaniform sensilla receive forces in the form of strain in the cuticle. Due to the orientation-dependent directional sensitivity of the sensilla, the direction of the acting forces can be distinguished and the insect knows how its exoskeleton is oriented in space (Zill et al. 1981, 2004). In addition to these three methods, there is also the option of using visual information. Although gravity cannot be perceived visually, the brightness gradient between the bright sky and the darker earth, which points contrary to the gravitational vector, can be used for spatial orientation (Nalbach and Hengstenberg 1994; Matthews and Matthews 2009; Monteagudo et al. 2017).

In many studies that have investigated the perception of gravity and spatial orientation of insects, the body or body parts of the insects are fixed in partly unnatural way while the animals were loaded or rotated about their longitudinal and transverse axes (Bässler 1965; Horn and Kessler 1975; Horn and Lang 1978; Horn and Bischof 1983; Horn and Föller 1985; Nalbach and Hengstenberg 1994). The fixation of the animals however could lead to an altered behavioral response. The evaluation of the experiments is limited to the response of individual body parts and does not take into account the whole-body reactions and possible interactions between fixed and movable body parts. We here present a new method of 3D escape to study the spatial orientation behavior. This method is of high behavioral significance, especially for small animals that dwell on the ground and in holes and therefore can be easily submerged and disorientated in lose substrate. A situation like this requires fast and efficient recovery by orientating the body toward the gravitational vector and moving upward. In our novel setup we therefore fully submerged crickets (*Acheta domesticus*) in a lightweight substrate and disorientated the animals using fast rotations.

The setup was validated based by comparing our results to research findings of Horn and Bischof (1983) and Horn and Föller (1985), who had identified three subsystems that contribute to gravity perception as they rotated fixed crickets while evaluating compensatory head movements in their research on crickets (*Gryllus bimaculatus*). These include club-shaped sensilla on the cerci as specialized gravity receptors, a gravity-sensitive structure in the antennae, which is thought to be located between the first (scape) and second (pedicel) antennal segments, and a gravity-sensitive structure in the legs, which is attributed to the proprioceptive hair plates.

## Material and methods

### Experimental animals

Adult male and female house crickets (*Acheta domesticus*, Reptilienkosmos, Viersen, Germany) were chosen as test animals, as their gravity perception have been comparatively well researched and crickets have numerous gravity-sensitive sensory structures that are suitable for extensively validating our methodology. The animals were kept in groups of up to 50 specimens at room temperature (21.5 ± 0.5 °C) and a humidity of 61 ± 4% with a day–night cycle of 12 h. The insects were fed fresh food ad libitum. Crickets with missing legs, antennae or cerci, or animals with visible damages or deformities were excluded from the experiments.

### Experimental setup

**Fig. 1:**
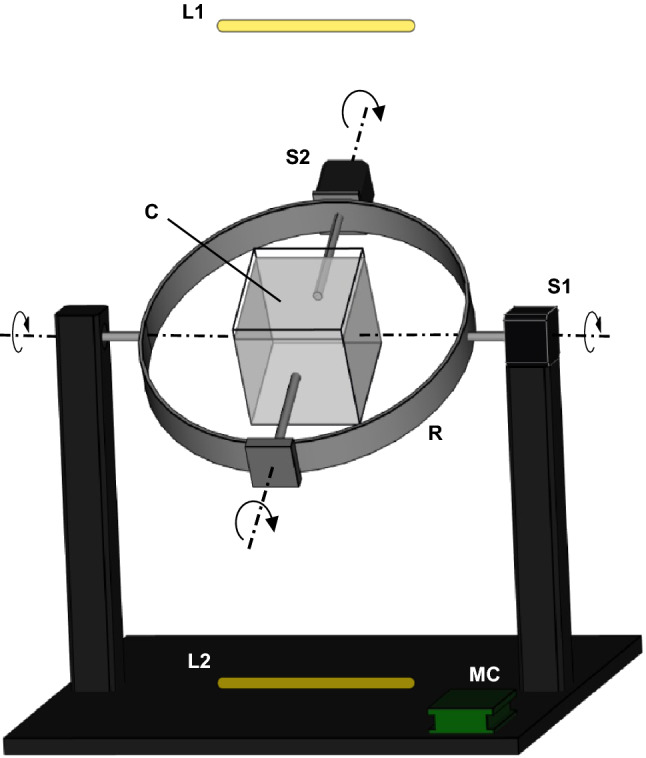
Experimental setup. Shown in black is the wooden construction on which the stainless-steel ring (R) is mounted. In the center of the ring a transparent cube (C) filled with granules is mounted. The two perpendicular axes of rotation are shown by the dashed lines. The rotation is controlled by stepper motor (S1, S2). The arrows indicate the direction of rotation. The stepper motors are individually controlled by a multicontroller with motorshield (MC). The position of the light irradiation can be varied by two separately switchable daylight sources (L1, L2). Not shown for clarity is the darkroom made of black cardboard, which houses the setup to avoid unwanted light irradiation, and three mirrors to observe all sides of the cube simultaneously

To disorientate the insects relative to the earth’s gravitational vector, a custom-made gimbal-like experimental setup was built from two movable parts (Fig. 1). A transparent cube (*C*: 100 × 100 × 100 mm) made of acrylic glass (HB Präsentationssysteme oHG, Niebüll, Deutschland) was placed in the center of a metal ring (*R*) with an outer diameter of 300 mm. The cube was filled with 95.5 g artificial snow (Rayher Hobby GmbH, Laupheim, Germany). Preliminary experiments showed that this material was the suitable substrate replacement, allowing for movement of completely buried individuals. Denser materials such as sand or sawdust were tested, however were too heavy and thus might trap and stress the animal unnecessarily. In addition, the selected artificial snow is significantly more translucent in order to examine possible effects of light on the orientation behavior of the animals. Two stepper motors (S1: Usongshine 17HS4401S; S2: Usongshine 17HS4023, Shenzhenshi Y.G. Electronics co., LTD., Shenzhen, China) allowed a precise rotation of the ring and the cube separately around two perpendicular axes of rotation. Mechanical stress to the system and the animals was reduced by linear acceleration and deceleration of the motors. To avoid unwanted light stimuli, the setup was surrounded by a cardboard housing. Two daylight LED (Lucky Reptile Mini Light Strip LED, Waldkirch, Germany) were placed at a distance of 295 mm vertically above (L1) and below (L2) the cube to determine a possible effect of light on insect gravity orientation.

At the beginning of each experiment the crickets were inserted head first through a glass tube with an inner diameter of 11 mm. The tube was inserted 5 cm into the artificial snow from the top center of the cube. This method of insertion ensured reproducibility, with all animals starting in the same initial position in the center of the cube. At this distance from the surface, the substrate removed approximately 95% of the incoming LED light, leaving a typical brightness of 13 lx for the crickets to detect. The dorsal–ventral orientation of the insect within the setup was randomized.

After insertion, the glass tube was removed; the lid of the cube immediately closed and the rotation of the cube around the two axes was started. The outer ring was rotated clockwise four full revolutions at a speed of 0.75 rps, while the cube was rotated counterclockwise two revolutions at 0.375 rps (10.6 s total trial time). With these settings, each side of the cube was oriented in each spatial direction for equal proportions of time per pass. The speed and number of rotations were chosen based on preliminary experiments to achieve disorientation of the insects with at the same time a minimum of stress for the animals. Longer spinning times or faster rotation did not show any effect on our results.

Within our experiments two parameters were varied. The end position of the cube either corresponded to the start position (0°) or the cube stopped rotated by 90° counterclockwise of its starting position (“end position 90°”). In addition, the experiments were repeated with illumination from either above or below. Although preliminary tests with some individuals showed no learning effects or even exhaustion of the animals, insects were only used once for an experiment within 24 h to exclude any habituation effects.

**Fig. 2:**
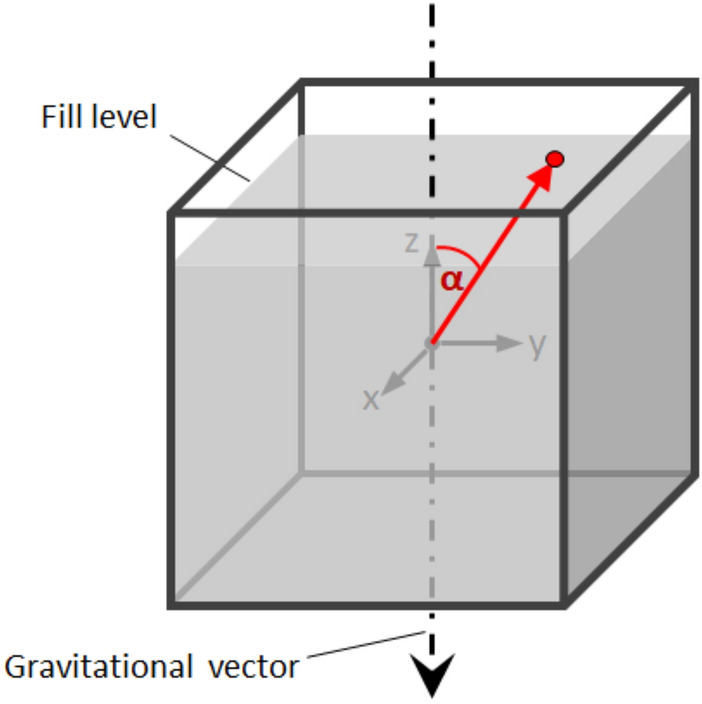
Schematic representation of the transparent cube filled with substrate. The starting position of the animals is represented by the origin of coordinates in the center of the cube. The gravitational vector is drawn in relative to this coordinate system. The red dot marks the place where the animal escaped. The trajectory is approximated by a vector (red arrow) between the start and end position. *α* is the angle between this vector and the Z-axis of the setup and thus the angular deviation to the gravitational vector, which in turn differentiates the accuracy

After stopping the rotation, the time each insect took to dig out (“excavation time”) was recorded, as well as the position of the cube at which an animal first broke through the surface of the substrate. These coordinates were used to calculate the trials respective angular deviation to the gravitational vector (Fig. 2) and determine the sensory performance of the crickets (Lindauer and Nedel 1959; Markl 1963).

Preliminary observations showed that insects not escaping within 10 min after stopping the rotation stayed immobilized at the starting position indefinitely. These trials were marked as “immobile” dropouts. Only experiments in which the insect escapes “upward” from the granules within 10 min by negative gravitaxis (angular deviation < 90°) were recorded as “successful”. Animals which escaped within 10 min, however, “downward” by positive gravitaxis (angular deviation > 90°) were recorded as “failed”. As the animal’s dorsal–ventral orientation was randomized during the experiments, the rotation of the location vector around the z-axis is not relevant for the analysis.

### Treatments

Horn and Bischof (1983) identified gravity sensitive substructures of crickets in their antennae, cerci and legs. To determine their respective sensory performance, we selectively deactivated these mechanosensitive receptor structures and several combinations of structures (see Table 1).

To facilitate preparation, insects were kept for 90 s at − 11 °C, which induced sufficient torpor for the preparation. All preparations were carried out using a stereo microscope (Stemi DV4, Carl Zeiss AG, Oberkochen, Germany). Insects were then allowed a recovery period of at least 72 h after treatment. As reference group we used crickets without any manipulation.

**Fig. 3:**
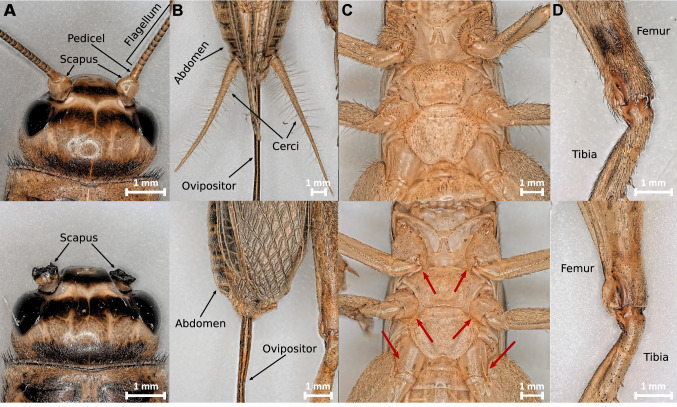
Overview of experimental treatments on gravitational sensors. **a** Dorsal view of the preparation of antennae on house crickets (*Acheta domesticus*). Above, an unprepared adult female whose antennae are complete. Below, an adult male with antennae removed below the pedicel in the middle of the scapus. **b** Dorsal view of the manipulation of club hairs on house crickets by ablation of cerci. Above, the abdomen of an unprepared adult female. Clearly recognizable are the cerci and the ovipositor. Below the abdomen of an adult female with completely removed cerci. **c** Dissection of the proprioceptive hair plates at the coxal joints. Above, the ventral view of an adult male with hair plates intact. Below, an adult male after removal of the proprioceptive hair plates at the coxal joints. The proprioceptive hair plates are located in the joint spaces, in addition to the proprioceptors, some tactile bristles were also removed. Red arrows indicate inaccessible areas where scattered hair sensilla remain. **d** Proprioceptive hair plates at femur–tibia joint. Above, representatively shown by right hind leg of an adult male. Below, a femur–tibia joint after removal of the proprioceptive hair plates

In the test group “*Antenna*” both antennae were cut off with a scalpel in the middle of the scape, so that the pedicel, which already has been associated with gravity perception in some insects, was also removed (Fig. 3A). In the test group “*Cerci*” the club shaped sensilla on the cerci of the crickets were also removed by completely ablating both cerci with a scalpel at their base on the abdomen (Fig. 3b).

Proprioceptive hair plates are thought to play an important role as gravity-sensitive structures in the legs. To experimentally determine the role of these receptors, the hair plates at the coxal joints (Fig. 3c) and at the femur–tibia joints (Fig. 3d) of all six legs were carefully removed. This was done using a toothpick wrapped in double-sided adhesive tape and carefully rolled over the hair plates, pulling out the hair sensilla. In places that were difficult to access, the hairs were also shaved off with a scalpel. Due to experimental limitations only 80–90% of the proprioceptors could be removed from the coxal joint without injuring the animals. The hair plates at the femur–tibia joint, on the other hand, could be removed almost completely. The shaved crickets were listed in the test group “*Hair plates*”.

To assess the detailed relative hierarchy and interactions of the sensory structures, we applied additional sensory ablations to groups which already had treatments, after having tested these previously. Animals with combined treatments were listed in the groups “*Antennae* + *Hair plates*” (crickets without antennae and without hair plates near the leg joints), “*Antennae* + *Cerci*” (animals with amputated antennae and removed cerci), “*Hair plates* + *Cerci*” (crickets with removed hair plates on the legs and amputated cerci) and “*Antennae* + *Hair plates* + *Cerci*” (animals on which all preparations were performed). Group sizes vary between 27 and 32 animals, these different group sizes are caused by excluded individuals or crickets that died.

### Statistics

Statistics were calculated using RStudio (V1.3.1093, RStudio PBC, Boston, USA). Since no parametric value distribution was found, the main effects were investigated with a nonparametric multifactorial ANOVA according to Puri and Sen (1985). This is a generalized extension of the Kruskal–Wallis test, which enables a multi-factorial analysis of nested or hierarchical sample structures. Samples were evaluated with regard to possible mean differences (Lüpsen 2021). In a first step, preparation, end position, and light irradiation were investigated as main factors in this nonparametric multifactorial ANOVA of pooled samples, to detect possible interactions between treatments and experimental parameters. The same was performed in a second step on pooled samples with single treatments (antennae, cerci, hair plates) as three main factors. This allowed us to detect possible factor contributions and interactions between the treatments. The post hoc analysis was performed with pairwise group comparisons by the pairwise Wilcoxon test (Sachs 1984). A value of at least *p* < 0.05 was considered as significant (*). The number of samples included in the analysis is indicated in the respective figures by *n*.

## Results

To initially assess the general variance of the results and of an individual insect, preliminary tests were performed measuring the responses of an individual insect in 20 consecutive measurements over a period of 24 h. This series of measurements also serves as animal controls. The individual insect successfully escaped upward to the surface of the granulate by negative gravitaxis (angular deviation < 90°) in all measurements within 10 min. The individual showed a mean angular deviation to the gravity vector of 29° ± 15° with an interquartile range of 27° and a mean “excavation time” of 88 s ± 22 s with an interquartile range of 30 s.

### Rate of success

Table 1 shows the rate of success for the mobile animals of all treatment groups. All specimens of the test groups “Reference”, “Antennae” and “Antennae + Hair plates” escaped successfully “upwards” regardless of their treatment and the experimental parameters. Also, in the groups “Hair plates”, “Cerci”, “Hair plates + Cerci” and “Antennae + Hair plates + Cerci” all of the animals that show a reaction successfully escaped by negative gravitaxis. However, there are some animals in these groups that do not respond and remain in their starting position during the experimentation time and are considered as immobile dropouts. With ten immobile animals, the most dropouts were in the group with removed cerci (34% dropouts), followed by seven animals in the group with additionally removed hair plates (23% dropouts). Three immobile animals are to be deplored in the groups with removed hair plates (9% dropouts), as well as the group with all mentioned manipulations (10% dropouts). The test group “Antennae + Cerci” was the only group where four individuals showed active movement and escaped toward the “wrong” direction (positive gravitaxis). These insects were the only individuals whose attempt was recorded as “unsuccessful” due to their angular deviation to the gravitational vector. Overall, 84% of the mobile animals in this group still escaped successful. With five immobile animals, this group had a dropout of 17%.

**Table 1:** Rate of success of insects with different treatments

Treatment	Group size	Mobile			Immobile drop outs
		Successful	Failed	Rate of success	
Reference	27	27	0	100%	0 (0%)
Antennae	28	28	0	100%	0 (0%)
Hair plates	32	29	0	100%	3 (9%)
Cerci	29	19	0	100%	10 (34%)
Antennae + Hair plates	28	28	0	100%	0 (0%)
Antennae + Cerci	30	21	4	84%	5 (17%)
Hair plates + Cerci	31	24	0	100%	7 (23%)
Antennae + Hair plates + Cerci	30	27	0	100%	3 (10%)

The number of individuals in the corresponding experimental group is listed under "Group size". The table distinguishes between "Mobile" animals that show a response and "Immobile" animals that do not respond at all and remain in their starting position for the first 10 min. “Mobile” animals are divided into “Successful” and “Failed”. Trials in which an animal excavated upward within the acrylic cube by negative gravitaxis (angular deviation < 90°) within 10 min were considered successful. The percentage of successful trials of the mobile part in a group is listed under “Rate of success”. "Failed" determines the number of failed attempts in which animals escaped downward by positive gravitaxis, i.e., animals with a proven modified response. Immobile animals are treated as dropouts. Group sizes vary as individuals were excluded from the study due to loss of limbs, death, etc.

### Angular deviation

Measuring the angular deviation of the escaped insect from the earth’s gravitational vector allowed us to compare the effects of the respective treatments. Larger deviations can be seen as a worse performance than smaller deviations from the zero axes.

The angular deviation to the “true” gravitational vector of insects escaping from a 90° rotated cube was not significantly different from insects escaping from a 0° rotated cube (*X*^2^ = 0.4006; *p* > 0.05). This shows that the insects were able to experience the gravitational vector during the experiment and perform a directed escape movement within the box. The sex of the tested insects also had no significant effect on the angular deviation (*X*^2^ = 0.0514; *p* > 0.05). For the body orientation this is also true depending on the treatment of the animals (*X*^2^ = 0.6467; *p* > 0.05). The light-direction (top/bottom) had no significant effect on the angular deviation across the entirety of the test groups (*X*^2^ = 0.5065; *p* > 0.05), as well as in dependence of the treatment (*X*^2^ = 1.8821; *p* > 0.05). A detailed statistical analysis shows an overall highly significant effect of the treatment on the angular deviation (*X*^2^ = 23.7455; *p* < 0.001). The statistical evaluation on pooled samples in a factorial manner with the single treatments as three main factors (cerci, antennae, hair plates) provides a highly significant effect of the ablation of cerci on the angular deviation (*X*^2^ = 19.9910; *p* < 0.001). No significance can be demonstrated for the removal of the antennae over the pooled sample (*X*^2^ = 0.7392; *p* > 0.05). However, for the hair plates a significant effect can be observed over the entirety of the samples again (*X*^2^ = 4.9893; *p* < 0.05).

**Fig. 4:**
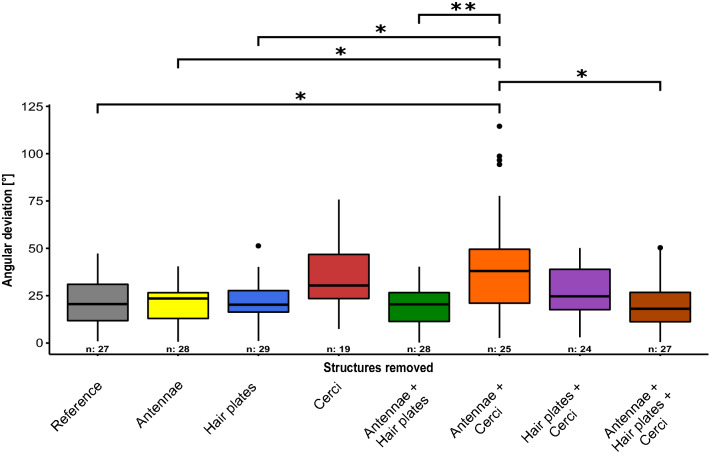
Angular deviation of the successfully escaped insects. Influence of the treatment of the test animals (*Acheta domesticus*) on the angular deviation [°] to the gravitational vector during excavation. The individual test groups are color coded depending on their treatment. Significant differences (*p* < 0.05) from pairwise group comparison are indicated by asterisks, *n* is the number of samples included in the evaluation. In particular the group with removed Antennae and Cerci showed significant differences to many other treatment groups

Figure 4 shows the angular deviation of all experimental groups along with the significances obtained in the pairwise group comparisons. For the test groups “*Reference*”*, *“*Antennae*”*, *“*Hair plates*”*, *“*Antennae* + *Hair plates*” and “*Antennae* + *Hair plates* + *Cerci*” average angular deviations from the earth’s vector were between 20° (*Antennae* + *Hair plates*) and 22° (*Hair plates*). Accordingly, these experimental groups do not differ significantly from each other.

In insects with removed cerci, we observed an average angular deviation of 34°. Insects with removed cerci and shaved hair plates on their legs showed an angular deviation of 27°. The group with “*Antennae* + *Cerci*” removed was the only treatment showing significant differences in angular deviation to groups with other preparations. The average angular deviation of this group with 44° was more than twice as large as the angular deviation of the reference group (22°, *p* < 0.05), the group with removed antennae alone (21°, *p* < 0.05), the group with antennae and hair plates removed (20°, *p* < 0.01) and the group with all treatments (20°, *p* < 0.05). Also, the average angular deviation of the animals with removed antennae and cerci was significantly larger than that of the test group with removed hair plates (22°, *p* < 0.05). Comparing group "Antennae + Cerci" with group "Antennae + Hair plates + Cerci", it is noticeable that ablation of hair plates has a significant effect (*p* < 0.05) when antennae and cerci were also removed.

### Excavation time

To further distinguish between accuracy (angular deviation) and possible other effects of the treatment we measured the excavation time for each insect reaching the outer side of the cube within a maximum of 10 min.

**Fig. 5:**
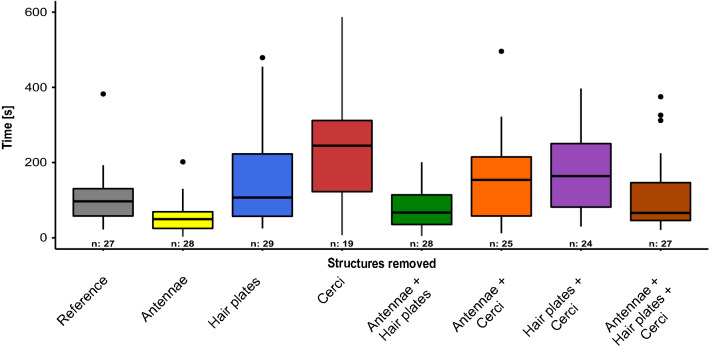
Excavation time. Influence of the treatment of the test animals (*Acheta domesticus*) on the time required for excavation [s]. The individual test groups are color coded depending on their preparation. n is the number of samples included in the evaluation. On average, insects with removed cerci needed the longest times to excavate, while animals without antenna were on average the fastest. For clarity, significances are shown in Table 2

The excavation times for different treatments are shown in Fig. 5, with results of the statistical pairwise group comparisons shown in Table 2. Again, across all groups neither the sex of the tested insects nor the body orientation had a significant effect on the excavation time (*X*^2^ = 2.0582; *p* > 0.05, *X*^2^ = 0.2588; *p* > 0.05). For the body orientation this is also true depending on the treatment of the animals (*X*^2^ = 0.9987; *p* > 0.05). There was also no significant effect of the light orientation on the excavation time (*X*^2^ = 0.6999; *p* > 0.05). The treatment of the experimental animals however had an overall highly significant effect on the time required for excavation (*X*^2^ = 24.0394; *p* < 0.01). The statistical multifactorial analysis on the pooled samples with the single treatments (cerci, antennae, hair plates) as main factors offered a highly significant effect of the cerci (*X*^2^ = 24.6867; *p* < 0.001) as well as of the antennae (*X*^2^ = 11.7287; *p* < 0.001) on excavation time. For the removal of hair plates, a strongly significant effect can be shown over the entirety of the samples (*X*^2^ = 8.1628; *p* < 0.01).

**Table 2:** Significance table with pairwise group comparisons for excavation time

	Antennae + Cerci + Hair plates	Reference	Antennae	Hair plates	Cerci	Antennae + Hair plates	Antennae + Cerci
Reference	n.s.						
Antennae	n.s.	*p* < 0.05					
Hair plates	n.s.	n.s.	*p* < *0.01*				
Cerci	p < 0.05	*p* < 0.05	***p* < 0.001**	n.s.			
Antennae + Hair plates	n.s.	n.s.	n.s.	n.s.	*p* < *0.01*		
Antennae + Cerci	n.s.	n.s.	*p* < 0.05	n.s.	n.s.	n.s.	
Hair plates + Cerci	n.s.	n.s.	***p* < 0.001**	n.s.	n.s.	*p* < 0.05	n.s.

For clarity, this table is supplementary to Fig. 5

*n.s.* indicates non-significant comparisons

A value of *p* < 0.05 was considered as significant (*), *p* < 0.01 (**) and *p* < 0.001 (***)

While insects from the reference group with all sensors intact took on average 106 s to excavate from the substrate, insects without antennae took only about half the time with only 54 s. Insects with removed cerci took the longest time of all test groups (248 s). In general, across all treatments, additional removal of the antennae showed a trend to reduce the excavation time. In contrast, treatments involving the removal of the cerci showed a trend toward prolonging the excavation time. Similar to the results of the angular deviation, insects with cerci removed (and all other sensors present) showed the longest average excavation time of all tested treatments (248 s), followed by the group with hair plates and cerci removed (184 s).

## Discussion

In this paper, we present a new method for studying the spatial orientation behavior of insects and verified its suitability for behavioral experiments on spatial orientation. Instead of characterizing locomotion of insects on for example tilted platforms (Lindauer and Nedel 1959; Markl 1962) or rotate tethered insects (Horn and Lang 1978; Horn and Bischof 1983; Horn and Föller 1985; Nalbach and Hengstenberg 1994), in our experimental setup the insect is fully submerged within a substrate in a small box. The box is rotated around two axes to disorientate the insect and simulate in vivo disorientation, such as a small earth slide, as closely as possible. Then, the insect's 3D escape from the substrate is evaluated in terms of a whole-body response. Using this setup in combination with ablation of various sensory structures and characterizing resulting changes in escape behavior, allows to identify structures that contribute to the spatial orientation of the insect.

To assess the suitability of our methodology, we performed several ablation experiments and compared our results to literature on gravitational sensors, in particular the comprehensive study by Horn and Bischof (1983). The results of the biomechanical experiments performed in this study allow to estimate the relative role and interaction of various gravitational sensitive organs in the house cricket. Measuring the overall success and the angular vector at which the insects escaped the substrate allowed us to estimate the general performance and the respective accuracy of their sensor system and to describe the relative interaction between the sensors. Taking into account the escape time allowed us to confirm trends from the angular deviation and to interpret cricket behavior and response. In general, our experimental setup was designed to resemble a natural situation as close as possible, where the animals were for example buried by dislocated substrate and needed to orientate their bodies in respect to the gravitational vector to successfully escape the situation. Considerable preliminary work was performed to find a suitable combination of experimental parameters (type of substrate, illumination, rotational speed and duration) to perform these experiments on house crickets. For other insect species these parameters might need to be adjusted accordingly.

### Rate of success

The “success rate” measured in our experiments provides an overview of whether the tested animals are still showing a response (mobile/immobile) and if the response is clearly manipulated (successful/failed) with appropriate treatment. Immobile animals are treated as dropouts as there might be several reasons for this behavior not necessarily associated with a loss of gravity perception. It is conceivable, for example, that immobile animals simply need more time to orientate themselves spatially for their locomotion than the intended measurement time. Horn and Bischof (1983) showed in their experiments that the crickets were reluctant to move by themselves, so that they had to be motivated to walk by touching their wings. This natural behavior might also be an explanation for immobile animals. Behavioral changes as a result of the preparation cannot be completely ruled out either. The largest percentage of immobile dropouts can be found in groups with removed cerci. In addition to the club-shaped sensilla, crickets have several other sensory structures on their cerci, including thin filiform mechanosensory hairs, which are even able to perceive slight airflows (Gnatzy and Schmidt 1971; Miller et al. 2011). To mechanical stimuli of these filiform hairs the cricket reacts with escape behavior (Ifere et al. 2022). If the cerci including filiform hairs, however, are removed, these escape reflexes may not be induced, the animal might not show any escape reaction and could be recorded as immobile. Regarding gravity perception, Horn and Bischof (1983) found no difference between crickets with completely removed cerci and crickets in which only the club-shaped sensilla were removed. For future studies with this setup, it is worth performing more precise and localized ablations, for example by just removing the club-shaped sensilla. This way, other sensory structures on the cerci, such as the filiform hairs, are preserved and the submerged crickets presumably retain their escape reflexes, so that immobile dropouts might be reduced or even avoided altogether.

With four individuals classified as “failed”, "Antennae + Cerci" is the only group in which animals deviate more than 90° from the gravitational vector. So, they react with a positive gravitaxis downward. It seems likely that at least these four animals are no longer able to orientate themselves spatially. Possibly the recovery time in these four animals was not sufficient to develop complex long-time adaptations to compensate the removed sensory structures. While Horn and Bischof (1983) found manipulated compensatory head movements when only the antennae or cerci were removed, no compensatory head movements were found in the combined ablation of antennae and cerci, as long as the legs were not loaded, and the crickets were denied their ability to perceive gravity. Given that our findings agree very well with the results of Horn and Bischof (1983), who also found largest effects for the combined ablation of antennae and cerci, the lower success rate of the animals without antennae and cerci in our setup are most likely a result of reduced orientation performance.

### Angular deviation

The angular deviation to the gravitational vector of the insects during excavation can be used to estimate the accuracy of gravity perception. Accordingly, the smaller the angular deviation to the gravitational vector, the greater the sensing performance. Lindauer and Nedel (1959) as well as Markl (1962) evaluated their behavioral experiments quite similar, which justifies the use of this type of measurement.

Our results show that animals of the reference group with all receptors intact dug out by negative gravitaxis regardless of their body orientation within the gravity field. In particular the successful escape of the insects excavating from the 90° tilted box show that the crickets did not simply dig back in the initial direction or even in a random direction, however oriented themselves actively and specifically to the gravitational vector. This confirms the ability to perceive gravitational direction and naturally also implies the existence of gravity-sensitive sensory structures.

Horn and Bischof (1983) showed that amputation of the antennae reduced compensatory head movements, which revealed a significant effect on gravity perception. In our setup the removal of the antennae alone has no effect on the angular deviation of the crickets to the gravitational vector, for the comparison of the group without antennae with the reference group as well as over the entirety of the pooled samples. The function of the antennae might be notably restricted if the crickets are buried within the substrate. Looking at the structures that could be responsible for the perception of gravity in the antennae of the crickets, it seems likely that these structures can only be used to a limited extent or not at all in our experimental setup. The Johnston organ, a vibration sensor in the second antennal segment, which detects airflow caused by sound (Göpfert and Robert 2002; Todi et al. 2004; Yack 2004) as well as gravity-induced deflections of the third antennal segment at the connection of the third to the second segment in Drosophila, requires free mobility between the segment joints (Kamikouchi et al. 2006; 2009). Horn and Kessler (1975) realized the active movement of the antennae most likely is a requirement for the function of the proprioceptive hair plates between the first and second antennal segments, which are associated with the perception of gravity in the antennae of crickets. Even if it has not yet been conclusively clarified where the gravity perception takes place in the antennae of insects, it is assumed that the antennae must be actively moving. The reason that we find a smaller influence of the antennae in the crickets' gravity perception than Horn and Bischof (1983) might therefore be due to the fact that the function of the gravity-sensitive structures in the antennae is disabled in submerged crickets. The antennae are most likely too thin and flexible to move against the granules. The required active movement of the antennae is no longer possible. In this case, the type of ablation would not have made any difference, since the function of the antennae is restricted anyway, both when the antennae are still available and when the antennae are removed. Admittedly, the complete ablation of the antennae is a very extreme treatment; however, for simplicity we consider the whole antenna as an integrated gravity-sensitive system, since it has not yet been finally clarified which structures in the antennae are affected by gravity. Although Horn and Bischof (1983) suspect that receptors between the scapus and pedicel contribute to the perception of gravity in the antennae, the antennae in the middle of the scapus were also amputated here. We chose the same ablation method to ensure comparability with their study. Furthermore, complete ablation represents more of a “naturally occurring damage”, since the loss of the entire multisensory organ seems more likely than the selective loss of individual microstructures as a result of an injury. It is possible that the limited function of the submerged antennae already leads to a loss of the reference group's perception of gravity, which would explain why we find no difference between animals without antennae and the reference, but Horn and Bischof (1983) do. This could also explain the high scatter of 15° of one individual without treatment, since burial already leads to losses in the function of the present but trapped antennae.

Although no effect was detected when comparing animals without cerci with the reference group, our study shows a trend that the group without cerci tends to show a higher angular deviation. On the other hand, a highly significant effect of the ablation of the cerci on the angular deviation can be seen within the pooled samples for the entirety of samples, with the amputation of the cerci leading to a larger angular deviation and, thus, a lower accuracy in the gravity perception. Behavioral changes that alter gravity perception as a result of the crude ablation of the cerci can be ruled out, since Horn and Bischof (1983) already showed that gravity perception is not affected by whether the club hairs are shaved, or the cerci are completely removed. In principle, possible changes in behavior as a result of the removal of the filiform hairs, which are also located on the cerci, are excluded in the form of immobile dropouts at this point in the analysis, since only mobile animals are considered. This is in good agreement with an earlier study by Horn and Bischof (1983) identifying the club-shaped sensilla located on the cerci as true gravity receptors. In their experiments, the amputation of the cerci resulted in a reduction of the compensatory head movements and an associated reduction in the gravitational sensing performance. Looking at the small size of the club-shaped sensilla, it becomes clear that these sensilla, even when buried, have enough space in the interspace of the granules to deflect along the gravitational vector and to provide information for spatial orientation.

In addition to the cerci and antennae, Horn and Föller (1985) also identified gravity-sensitive substructures in the legs of crickets, which elicit compensatory head movements during passive rotation. Gravity perception in the legs presumably occurs via the proprioceptive hair plates at the joints of the legs, whose involvement in gravity perception has already been shown for some insects (Bässler 1965; Lindauer and Nedel 1959; Markl 1962). The removal of the proprioceptors of the legs only showed no significant difference to the reference group in our setup. When comparing the group "Antennae + Cerci" with the group "Antennae + Hair plates + Cerci", however, it is noticeable that the removal of the proprioceptive hair plates on the legs has a significant effect, provided that antennae and cerci are also removed and lead to a reduction in the angular deviation during escape. In addition, by comparing the pooled sample of all groups with removed hair plates to all groups with hair plates present, a significant effect of the hair plate removal was observed. Nevertheless, this is still not an explicit proof of an involvement of the proprioceptive hair plates on the coxal joints and the femoral joints on the legs of *Acheta domesticus* in their gravity perception. As the proprioceptive hair plates detect relative displacement between moving body parts, there is a reason to believe that this mechanism does not work behaviorally when the animal is submerged and at least partially supported by the granule. It seems likely that the submerged animal in the immobile condition cannot extract spatial orientation information from the proprioceptive hair plates. This could justify why some of the animals with hair plates removed remain in the initial position. However, as soon as the animal moves in the granules, on the one hand forces have to be applied to push the granules, and on the other hand forces of gravity have to be counteracted during movement, whereby information about the gravitational vector can indeed be extracted from the proprioceptors in order to orient spatially. Furthermore, secondary effects due to the ablation of the hair plates, which may not affect graviception at all, cannot be ruled out completely. As is well known, the proprioceptors also serve to control movement, which is why altered locomotion behavior due to the ablation of hair plates on the legs would be conceivable. Wong and Pearson (1976) were able to document changed leg movements on individual legs of cockroaches by recording the muscle activities of walking animals before and after the ablation of the proprioceptive hair plates. It has not yet been clarified to what extent this change in movement also occurs in crickets and how strongly this affects their escape behavior and locomotion during excavation. In contrast to the club-shaped sensilla on the cerci, the proprioceptors are more sensitive to non-gravity stimuli. These include, for example, wind, mechanical contact and dynamic forces resulting from the walking movements, which can lead to proprioceptive reafferences (Horn and Föller 1985). Another way of perceiving gravity via the legs is the third mechanism (described above), in which campaniform sensilla perform distributed load sensing in the cuticle (Zill et al. 1981, 2004). This mechanism has not previously been associated with gravity perception in crickets. Also in this study, no preparations were made to manipulate the campaniform sensilla in the cuticle of the legs, since the functioning of proprioception is quite similar; however, it seems very likely that this mechanism of gravity perception could also be studied with our setup, if an active movement of the insects occurs.

In addition to the structures manipulated during this study, other gravity-sensitive structures must exist that allow crickets to receive the direction of the gravity vector. Even with all described manipulations accumulated, 100% of the mobile animals were still able to excavate successfully with an average angular deviation at a similar level as observed in unprepared animals. The strongest angular deviation is observed at the collective removal of antennae and cerci. This is consistent with the results of Horn and Bischof (1983), who also observed the largest effect in the collective ablation of antennae and cerci. Horn and Föller (1985) concluded that all gravity-sensitive subsystems interact at least at the motor level, since they produce equal responses to changing gravitational stimuli and their combined response is greater than of just one or two subsystems. Based on this conclusion, we would expect an even greater angular deviation if the proprioceptive hair plates were also removed. Thus, the accuracy of gravity sensing does not decrease as expected when an additional structure is removed in addition to the antennae and cerci, but actually increases. This indicates previously unexplained non-linear interactions between the gravity-sensitive sensory structures and additional gravity-sensitive structures that were not considered in our experiments. Due to the complexity of the interplay of the different sensory structures, no more precise statements can be made at this time, which justifies further research in this area.

### Excavation time

Interestingly, the ablation of the antennae (antennectomy) lead to an almost 50% decrease of excavation time compared to the reference group (Fig. 5). Antennectomy has been shown to significantly affect behavioral aspects of various insect species (Balakrishnan and Pollack 1997; Schütz and Dürr 2011; Sakura and Aonuma 2013). Simple observation of crickets with intact antennae shows that these individuals invest notable time for tactile exploration. Without antennae the respective sensory input is eliminated and other behavior such as gravitationally directed escape movements might dominate, which then could speed up the overall excavation time. Indeed, during our experiments we frequently observed that the crickets use their antennae to scan their surroundings for a few seconds before excavating completely. This behavior was not observed in experimental animals without antennae. Crickets with this manipulation thus excavated almost exclusively without interruption and without first extensively exploring their surroundings. Furthermore, insects without cerci required significantly more time to excavate (248 s) in comparison to the reference group (106 s). Simple observations of free running crickets show no noticeable difference between insects with and without cerci, which is why behavioral changes as a result of the preparation are very unlikely. It might be possible that buried insects without cerci need much more time to orient themselves in unfamiliar surroundings and to detect the gravitational vector. This would confirm our results of the angular deviation as well as the results of Horn and Bischof (1983) that the cerci play the major role in spatial orientation.

The meaningfulness of the excavation time for spatial orientation is significantly lower than the angular deviation or the rate of success, because many more factors influence this evaluation parameter. Thus, temperature, treatment, etc. can influence the behavior and thus the speed of excavation. However, the excavation time can be used to confirm trends that appear in the angular deviation or to interpret the orientation behavior and responds of the animals. If an animal or experimental group requires significantly more time, this could indicate that the animal needs more time to orient spatially because the sensory structures still present provide less accurate information, or that information from the remaining sensory structures must be evaluated against each other.

### Verification of the 3D-escape methodology

In general, the results obtained by our experimental setup and approach are in good agreement with the previously known results of Horn and Bischof (1983). Although Horn and Bischof (1983) determined greater effects of the individual ablations than could be demonstrated in our experiments, the greatest effects on gravity perception were observed in our experiments as well as in Horn and Bischof's (1983) experiments through the collective ablation of antennae and cerci. The smaller effect of the single ablations in our setup could indicate a lower accuracy of our results. However, the restricted function of especially thin, long and flexible structures such as antennae might also be a possible reason for the comparatively small effects that could be achieved with our setup. According to this, the function of the antennae is restricted in submerged animals, which is why a reduced gravity perception might already be existent in the reference group due to the failure of the antennae. An antennectomy would therefore have no effect, since the antennae are not fully functional in submerged animals anyway. The ablation of other structures, such as the cerci and hair plates, would therefore also have smaller effects than was observed by Horn and Bischof (1983). Since the reduced perception of gravity in the reference group due to our setup means that there are smaller differences between the reference group and groups of manipulated animals than in the case of Horn and Bischof (1983), where the reference animals' perception of gravity is not impaired. Nevertheless, the influence of the ablation techniques on the accuracy of the results should not be neglected. The possible lower accuracy of our results and the restricted function of individual sensory structures can be a disadvantage; however, this can be counterbalanced by some experimental advantages like the whole-body response and the natural situation represented by our setup. While previous studies investigated either the whole-body reaction of freely moving insects on, for example, tilted platforms (Lindauer and Nedel 1959; Markl 1962), or the reaction of individual body segments of fixed insects, in which a targeted disorientation of the animal or a targeted change in gravity took place (Horn and Bischof 1983; Horn and Lang 1978; Horn and Föller 1985; Nalbach and Hengstenberg 1994), both aspects are combined in our setup. In our setup, the evaluation of the whole-body reaction of untethered animals is possible in all spatial directions, while the animals can be disoriented in a very targeted manner at the same time, by separately or parallel varying parameters such as rotation angle and rotation speed, start and end position, duration of rotation as well as the angle and intensity of the light irradiation. With the whole-body reaction, especially interactions between different sensory organs can be investigated since no body parts have to be fixed. Furthermore, easy and straight forward adjustments or extensions of our methodology are possible. The granulate can be changed, for example, to reduce the influence of light (black granulate) or to increase it (transparent granulate). In addition, there is the possibility of manipulating acceleration forces in addition to gravity. Furthermore, it would certainly be interesting to observe the animal movements inside the substrate, in particular to be able to identify and analyze changes in behavior due to the ablation techniques that could be implemented, for example, by life X-ray images.

The presented novel 3D-escape paradigm thus represents a more comprehensive and potentially powerful alternative to the previously known methods for investigating spatial orientation. As our setup replicates a more natural situation where an animal might be buried and disorientated by dislocated substrate, we believe that this new experimental setup is of increased behavioral significance especially for an animal living on lose substrate and underground. Our results further confirm that the angular deviation to the gravitational vector as well as the rate of success can be used to evaluate the orientation performance and the accuracy of gravity perception. The excavation time can be used as a parameter to interpret the orientation behavior and reactions of the test animals or to confirm trends that are evident in the angular deviation.

## Conclusion

In this study, we present a new experimental setup for comprehensive investigation of spatial escape behavior of insects. This setup allows to record behavioral responses relatively close to natural conditions without the need of complex sample treatment. Since no fixation of the insect’s body or body parts is required, whole-body orientation can be evaluated to also take into account interactions between gravity-sensitive structures for spatial orientation. Our results are in very good agreement with previous studies and support that gravity perception of crickets (*Acheta domesticus*) is not based on a single type of sensor alone, but on a fusion of information of different cooperating sensory structures. This study shows that these sensory structures differ in their importance for gravity perception and achieve different gravity sensing performances, resulting in a relative hierarchical order. The club-shaped sensilla on the cerci were the most important in cricket’s gravity perception among the structures investigated in this study, and also achieved the greatest gravity sensing performance. Antennae also contribute to gravity sensing accuracy to a lesser degree than the club-shaped sensilla of the cerci. They seem beneficial to compensate for the failure of the cerci to a certain extent. The proprioceptive hair plates on the legs are probably of even less importance. In addition, also other exoskeletal mechanoreceptors might contribute toward gravity reception.
